# The effect of acceptance and commitment therapy on depression in parents of children with special needs: a meta-analysis

**DOI:** 10.3389/fpsyg.2025.1590489

**Published:** 2025-07-09

**Authors:** Wei Wang, Haoran He

**Affiliations:** ^1^Beijing Institute of Graphic Communication, Beijing, China; ^2^School of Physical Education and Training, Capital University of Physical Education and Sports, Beijing, China

**Keywords:** acceptance and commitment therapy, parents of children with special needs, depression, meta-analysis, meta-regression

## Abstract

**Purpose:**

This study aimed to evaluate the effectiveness of Acceptance and Commitment Therapy (ACT) in alleviating depressive symptoms among parents of children with special needs. Additionally, it examined the moderating effects of geographic and cultural contexts, intervention parameters, and types of children’s diseases.

**Methods:**

A systematic search was conducted in PubMed, Web of Science, and the Cochrane Library. Inclusion criteria were: (1) peer-reviewed studies published in English, (2) randomized controlled trials (RCTs), (3) ACT as the core intervention, (4) participation by parents of children with special needs, and (5) reporting of standardized effect sizes for depressive symptoms. A total of 12 studies (*n* = 746) met the eligibility criteria. A fixed-effects model was employed for the meta-analysis, and subgroup analyses were conducted based on geographic distribution, intervention duration, intervention parameters, and types of children’s diseases (neurodevelopmental disorders, chronic illnesses, or severe conditions).

**Results:**

ACT significantly reduced depressive symptoms (SMD = −0.36, 95% CI [−0.51, −0.22], *p* < 0.0001). Subgroup analyses indicated that the parents of children with neurodevelopmental disorders or chronic illnesses, the national context, and the frequency of intervention were key sources of heterogeneity in treatment outcomes.

**Conclusion:**

The synthesized evidence suggests that ACT is particularly beneficial for parents of children with neurodevelopmental disorders and chronic illnesses. A recommended intervention duration of 4–8 weeks (≥35 min per session, 1–2 sessions per week) is optimal, and the effect may be the most significant in areas with a well-developed welfare system. Future studies should prioritize the development of culturally adapted intervention modules and explore digital health platforms to enhance accessibility.

## Introduction

In recent years, advancements in medicine (Chowdhury et al., 2025), education (Andriani et al., 2024), and social welfare (Manuel, 2021) have heightened societal attention toward children with special needs. Due to chronic illnesses, developmental, behavioral, or emotional issues, these children require significantly more services than their peers (Zanello et al., 2015). However, parenting a child with special needs [e.g., autism spectrum disorder (ASD), chronic illness, or neurodevelopmental disorder] poses unique psychological challenges. Research has shown that parents of children with ASD often experience grief, denial, shock, and self-blame at the time of diagnosis, with their emotions gradually evolving into long-term stress and depression (Filipova et al., 2024). During the COVID-19 pandemic, 35.8% of mothers of children with ASD exhibited both anxiety and depression (Santiago et al., 2024), and another report indicated that 58% of parents of children with ASD experienced depressive symptoms, while 44.4% experienced anxiety symptoms (Pondé et al., 2023). Parents of children with chronic illnesses and neurodevelopmental disorders often experience elevated levels of anxiety, depression, and stress, which adversely affect their quality of life (Fäldt and Fängström, 2024; Ververidou et al., 2023; Wahab and Ramli, 2022). Furthermore, parents of children with conditions such as cerebral palsy or cancer endure persistent psychological burdens due to the long-term coordination of medical and educational resources (Bamber et al., 2023; Kouther et al., 2022; Zeng et al., 2023). However, some parents exhibit remarkable resilience and personal growth, often facilitated by religious faith, community support, and effective coping strategies (Nursanti, 2024).

Traditional cognitive behavioral therapy (CBT) has demonstrated specific efficacy in alleviating depressive symptoms in parents; however, it has limitations in addressing the unique pressures of isolation and the significant caregiving burden faced by families with children who have special needs (Gaviţa et al., 2011; McIlrae et al., 2010; Singh et al., 2023). Consequently, family-based integrated interventions have been proposed to meet the needs of these families more comprehensively (Compas et al., 2015). Among these interventions, ACT, categorized as a “third-wave” therapy, promotes psychological flexibility by emphasizing the acceptance of internal experiences rather than attempting to change them and encouraging individuals to act according to their values. ACT comprises six core processes: acceptance, cognitive defusion, present-moment awareness, self-as-context, values, and committed action (Petersen and Pimentel, 2024). Neurobiological evidence has indicated that ACT can enhance prefrontal cortex activity while reducing amygdala reactivity, thereby improving emotional regulation (Brehl et al., 2020; Messina et al., 2016). Its effectiveness is believed to stem primarily from enhanced cognitive flexibility and emotion regulation rather than from distinct mechanisms of action. Previous systematic reviews of ACT have concentrated mainly on children’s physical and psychological wellbeing (Swain et al., 2015), with a lack of comprehensive meta-analytic reviews focusing on the mental health of parents of children with special needs. Based on this background, the present study aimed to conduct a systematic literature review and meta-analysis to investigate the overall effect of ACT on depressive symptoms among parents of children with special needs, explore moderating factors, and compare treatment outcomes across different populations and backgrounds.

## Materials and methods

### Study design

This study was designed as a systematic review of RCTs and was prepared using the Preferred Reporting Items for Systematic Reviews and Meta-Analyses (PRISMA) guidelines (Moher et al., 2015). Before screening the search results, the protocol was registered with the International Prospective Register of Systematic Reviews (PROSPERO; registration number: CRD420250652175) and was conducted in alignment with the PRISMA statement.

### Study inclusion criteria

Studies were eligible if they were full-text, peer-reviewed controlled experiments published in English that enrolled parents (of any gender, ethnicity, or socioeconomic background) of children with special needs (Population), delivered Acceptance and Commitment Therapy (ACT) in at least one structured intervention session (Intervention), and compared ACT to either an inactive control (e.g., wait-list or no-treatment) or another conventional psychological or behavioral treatment (Comparison), with depressive symptoms assessed pre- and post-intervention using a validated, standardized depression scale (Outcome). Narrative reviews, preclinical studies, duplicate reports, editorials, opinion pieces, conference abstracts/papers, and other grey literature were excluded, and although systematic reviews and study protocols did not meet inclusion criteria, they were reviewed for background guidance and cited where appropriate (Study Design).

### Search strategy

This meta-analysis aimed to evaluate the effects of ACT on depression among parents of children and adolescents with special needs. A comprehensive search was conducted across the PubMed, Web of Science, PsycINFO, and Cochrane Library databases, concluding on February 9, 2025. The search utilized the following terms: (“Acceptance and Commitment Therapy” OR “ACT”) AND (“Depression” OR “Depressive symptoms”) AND (Parents OR Mothers) AND (“children with cerebral palsy” OR “children with CP” OR “children with Autism Spectrum Disorder” OR “children with autism” OR “children with chronic conditions” OR “children with chronic illness” OR “children with acquired brain injury” OR “children with hearing impairment” OR “children with asthma” OR “children with cancer” OR “special needs children”) AND (“Controlled Trial” OR “Randomized Controlled Trial” OR “RCT” OR “Clinical Trial” OR “Controlled Study” OR “Comparative Study” OR “Placebo-Controlled Trial”). Furthermore, the reference lists of the retrieved studies were examined to identify additional eligible studies.

### Study selection process

The retrieved records were imported into Zotero 7.0. After removing duplicates, two reviewers independently screened the titles and abstracts of the studies. Studies that did not meet the eligibility criteria were excluded. Full-text versions of all relevant studies were obtained and further assessed for eligibility. To minimize bias in the selection process, any disagreements regarding study inclusion were resolved through consultation with a third independent reviewer. Data extraction from each study was performed independently by two reviewers, with any discrepancies resolved by consulting the third reviewer.

### Data synthesis

All meta-analyses were conducted using the R software environment’s meta, metaphor, diameter, and ggplot2 packages. For continuous outcome variables, either the mean difference (MD) or the standardized mean difference (SMD) was employed as the effect size, depending on the consistency of the measurement tools across studies. The SMD was calculated using Hedges’ g, interpreted as small (g = 0.3), medium (g = 0.5), and large (g = 0.8) effect sizes (Nakagawa and Cuthill, 2007). Pooled effect sizes were computed by weighting the inverse of the variance of each study’s effect size. The overall effect was primarily estimated using the DerSimonian-Laird random effects model; however, a fixed-effects model was also considered when heterogeneity was low. Heterogeneity was assessed using the chi-square test (with *p* < 0.10 considered significant) and the I^2^ statistic (with I^2^ > 50% indicating substantial heterogeneity) (Lau et al., 1997). A funnel plot was initially constructed to detect potential publication bias, followed by a visual inspection based on each trial’s effect size and standard error. Egger’s regression test was also employed to evaluate further funnel plot asymmetry, supplemented by a trim-and-fill method for sensitivity analysis (Hong et al., 2020). Outlier studies that exerted undue influence on the model were identified using standardized residuals (|z| > 2.5) and Cook’s distance (>3 times the mean) (Viechtbauer and Cheung, 2010). Furthermore, subgroup analyses, sensitivity analyses, and influence diagnostics (utilizing functions such as Metainf and Influence Analysis) were performed in R to explore the impact of individual studies on the overall effect and the robustness of the results. Meta-regression analyses were conducted using a random effects model based on the restricted maximum likelihood method (REML) to assess potential moderating effects, with bubble plots generated to visually illustrate the relationship between moderator variables and effect sizes (Van Houwelingen et al., 2002). To ensure comparability across different measurement tools, outcome scores from varying scales were converted to SMDs using the following formula: $\mathrm{SMD}=\frac{M_{\text{Intervention}}-M_{\text{Control}}}{{\mathrm{SD}}_{\text{Pooled}}},\;\;{\mathrm{SD}}_{\text{Pooled}}=\sqrt{\frac{(n_{1}-1){\mathrm{SD}}_{1}^{2}+(n_{2}-1){\mathrm{SD}}_{2}^{2}}{n_{1}+n_{2}-2}}\;$ (Hedges and Olkin, 2014).

### Risk of bias (quality) assessment

The quality of each included study was assessed using the Cochrane Risk of Bias tool for randomized trials, which evaluated several domains: random sequence generation, allocation concealment, blinding of participants and personnel, blinding of outcome assessment, incomplete outcome data, selective reporting, and other biases (Higgins et al., 2011). Two independent authors conducted the quality assessments, and a third reviewer resolved disagreements. Furthermore, the overall evidence was visualized and analyzed using EVDMAP,^1^ providing an intuitive presentation of the distribution and quality of the studies, thereby enhancing the overall understanding of the evidence base (Miake-Lye et al., 2016).

## Results

### Study selection

**Figure 1:**
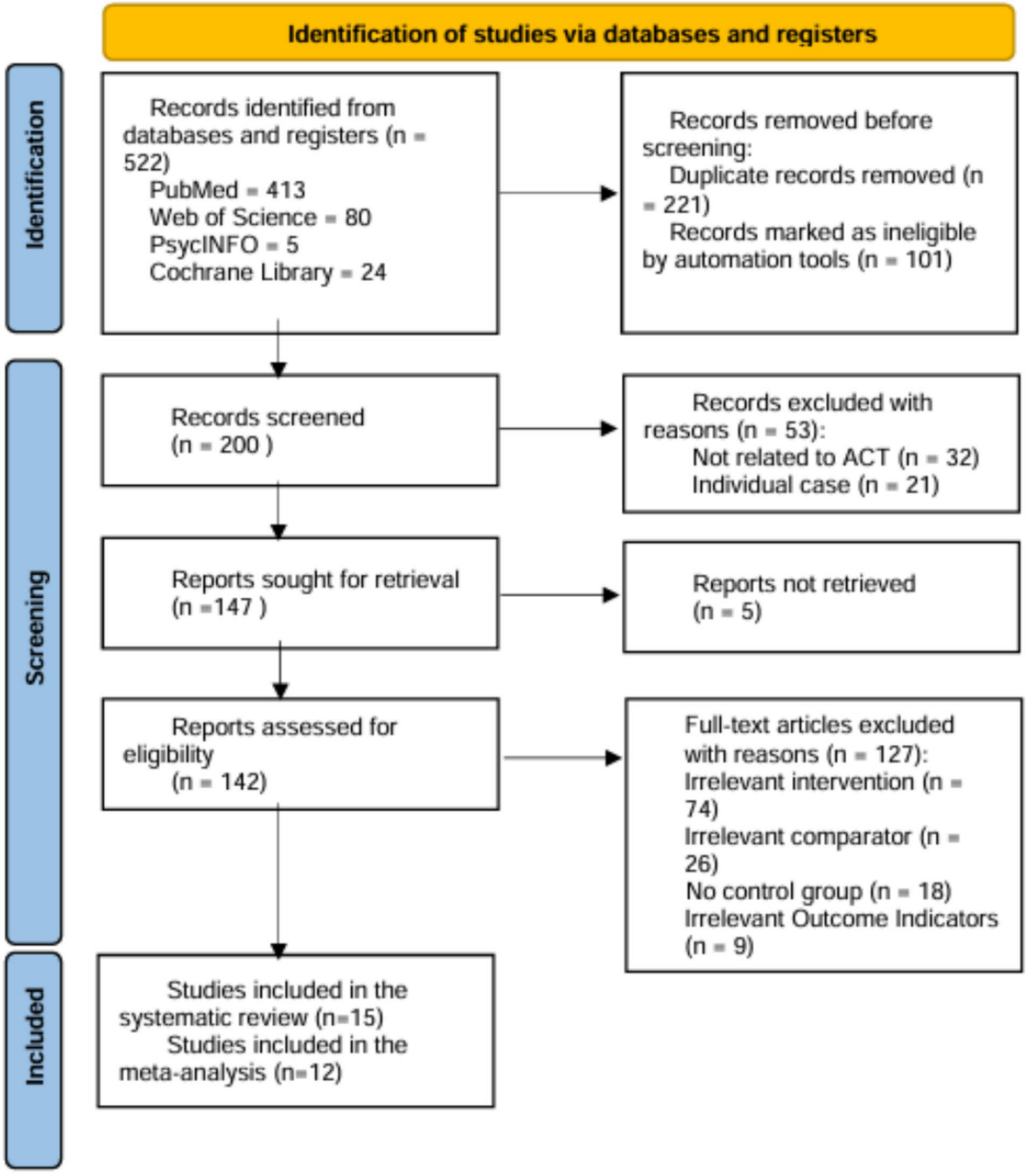
Flow diagram of the selection process. Three of the 15 studies in the systematic review were excluded from the meta-analysis due to heterogeneity and bias assessment.

Initially, 522 records were retrieved from the four databases, from which 221 duplicates were removed. After screening the titles and abstracts of 200 documents, 53 studies were excluded for failing to meet the inclusion criteria. The remaining 147 studies underwent a full-text review, excluding 127 for various reasons (as detailed in Figure 1). Ultimately, 15 studies were included in the systematic review (Brown et al., 2014; Chong et al., 2019; Çiçek Gümüş and Öncel, 2023; Douma et al., 2021; Gharashi et al., 2019; Hahs et al., 2018; Joekar et al., 2016; Joosten et al., 2024; Lappalainen et al., 2021, 2024; Maughan et al., 2024; RashidiFard et al., 2024; Sairanen et al., 2019; Whittingham et al., 2016, 2022).

### Risk of bias of included studies

**Figure 2:**
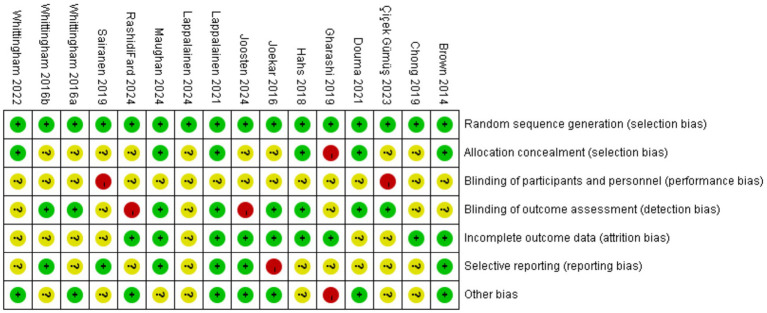
Risk of bias summary.

**Figure 3:**
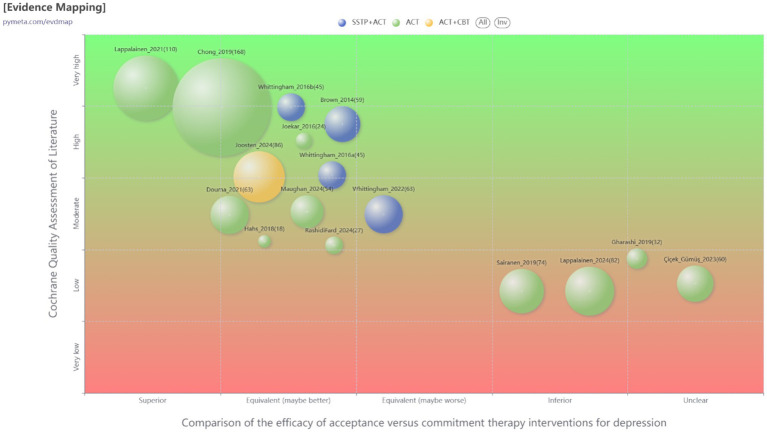
Evidence mapping of ACT interventions for depression.

All included studies adequately described the generation of random allocation sequences and were assessed to have a low risk of selection bias associated with random sequence generation. Six trials explicitly reported details regarding allocation concealment and were rated as having a low risk (Hahs et al., 2018; Lappalainen et al., 2021; Maughan et al., 2024; Douma et al., 2021; Brown et al., 2014; Whittingham et al., 2022). At the same time, the remaining studies lacked sufficient detail and clarity, resulting in unclear or high-risk classifications. Regarding performance bias, the nature and number of personnel involved in the studies made it challenging for blind coaches or researchers. Nevertheless, in studies assessing the impact of ACT on depression among parents of children and adolescents with special needs, the blinding of outcome assessments was critical. Attrition and reporting biases were significantly influenced by five studies (Whittingham et al., 2016; Douma et al., 2021; Lappalainen et al., 2024; Whittingham et al., 2022; Çiçek Gümüş and Öncel, 2023). Figures 2, 3 illustrate a summary and evidence map of the risk of bias, respectively. Detailed information is provided in the Supplementary Table 1.

### Study characteristics

The main characteristics of these studies are detailed in Table 1. Among the included randomized controlled trials, 15 studies (Hahs et al., 2018; Sairanen et al., 2019; Lappalainen et al., 2021; Maughan et al., 2024; Joekar et al., 2016; Lappalainen et al., 2024; Gharashi et al., 2019; Douma et al., 2021; Chong et al., 2019; RashidiFard et al., 2024; Çiçek Gümüş and Öncel, 2023) focused on the effects of ACT on the mental health of parents with children suffering from various conditions, including ASD, hearing impairment, pediatric acquired brain injury, cerebral palsy (CP), and chronic illness. Four studies (Brown et al., 2014; Joosten et al., 2024; Whittingham et al., 2016, 2022) examined the additional benefits of combining ACT with other interventions, such as the Stepping Stones Triple P or CBT. Most interventions lasted between 6 and 13 weeks, with individual sessions ranging from 35 to 180 min. The main characteristics of these studies are presented in detail in Table 1.

**Table 1:** Characteristics of the studies in the systematic review and meta-analysis.

Author/year	Author’s country	Research design	Sample size (T/C)	Age range	Subject type	Intervention design (T/C)	Intervention protocol	Evaluation tools/content
Whittingham et al. (2016)	Australia	RCT	45/37	38.73 ± 7.21	Parents of children with a diagnosis of CP	Stepping Stones Triple P and ACT/wait-list control group	120 min/session, 2 times/week, 6 weeks	DASS
Whittingham et al. (2016)	Australia	RCT	45/37	38.73 ± 7.21	Parents of children with a diagnosis of CP	Stepping Stones Triple P and ACT/Stepping Stones Triple P	120 min/session, 2 times/week, 6 weeks	DASS
Hahs et al. (2018)	America	RCT	18/13	45.5 ± 6.14	Parents of children with ASD	ACT/Control group	120 min/session, 2 times/week, 1 week	BDI-II
Sairanen et al. (2019)	Sweden	RCT	74/49	34.8–51.2	Parents whose children have chronic conditions	Web-based ACT/Control group	35 min/session, 1 time/week, 10 weeks	DASS
Lappalainen et al. (2021)	Finland	RCT	110/103	40.1 ± 6.69	Parents of children with chronic conditions	Supported iACT/Self-help ACT	45 min/session, 1 time/week, 13 weeks	PHQ-9
Maughan et al. (2024)	Canada	RCT	54/45	48.34 ± 8.79	Parents of autistic children, youth, and adults	Group-based ACT program/Waitlist group	180 min/session, 3 times/week, 7 weeks	DASS
Joekar et al. (2016)	Iran	RCT	24/24	34.96 ± 4.26	Mothers of children with high-functioning autism	ACT/Treatment as usual	90 min/session, 1 time/week, 8 weeks	DASS
Lappalainen et al. (2024)	Finland	RCT	82/73	40.1 ± 6.69	Parents of children with chronic conditions	Supported iACT/Self-help ACT	45 min/session, 1 time/week, 10 weeks	PHQ-9
Brown et al. (2014)	Australia	RCT	59/24	39.14 ± 1.88	Parents with pediatric-acquired brain injury	ACT + Standardized Stepping Stones Triple P/Care as usual	90 min/session, 1 time/week, 10 weeks	DASS
Gharashi et al. (2019)	Iran	RCT	32/32	29.31 ± 4.47	Parents with a child with a hearing impairment	ACT/Control group	90 min/session, 4 times/week, 2 weeks	DASS
Douma et al. (2021)	Netherlands	RCT	63/60	30.21–59.07	Parents of children with a chronic illness	ACT/Control group	90 min/session, 1 time/week, 6 weeks	HANDS
Joosten et al. (2024)	Netherlands	RCT	86/77	41.9 ± 7.5	Parents of children with cancer	CBT + ACT/Control group	120 min/session, 1 time/week, 7 weeks	PROMIS/DT-P
Whittingham et al. (2022)	Australia	RCT	63/*	30–45	Parents of children with a diagnosis of CP	Stepping Stones + ACT/waitlist control group	120 min/session, 1 time/week, 10 weeks	DASS
Chong et al. (2019)	China	RCT	168/88	38.40 ± 5.90	Parents of children with asthma	ACT/Control group	90 min/session, 2 times/week, 4 weeks	DASS
RashidiFard et al. (2024)	Iran	RCT	27/27	20–41	Mothers of individuals with ASD	ACT/Control group	90 min/session, 1 time/week, 8 weeks	BDI-II
Çiçek Gümüş and Öncel (2023)	Turkey	RCT	60/60	36–51	Parents with special needs	ACT/Routine training sessions	60 min/session, 1 time/week, 6 weeks	DASS

RCT, Randomized Controlled Trial; T, Intervention Group; C, Control Group; ACT, Acceptance and Commitment Therapy; iACT, Internet-based Acceptance and Commitment Therapy; CBT, Cognitive Behavioral Therapy; DASS, Depression Anxiety Stress Scales; BDI-II, Beck Depression Inventory – Second Edition; PHQ-9, Patient Health Questionnaire – 9; HADS, Hospital Anxiety and Depression Scale; PROMIS, Patient-Reported Outcomes Measurement Information System.

### Meta-analysis

**Figure 4:**
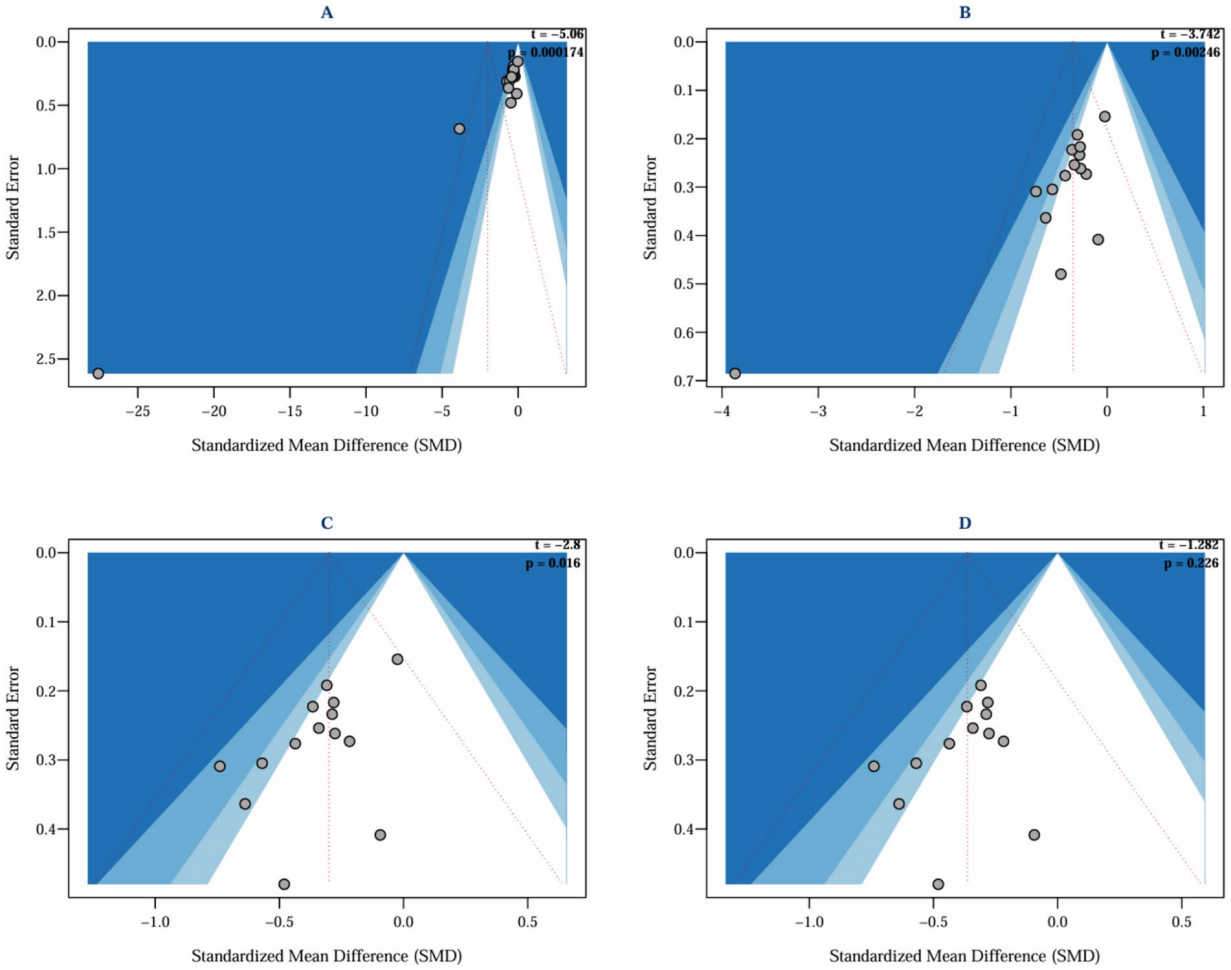
Funnel plot and Egger’s publication bias test.

**Figure 5:**
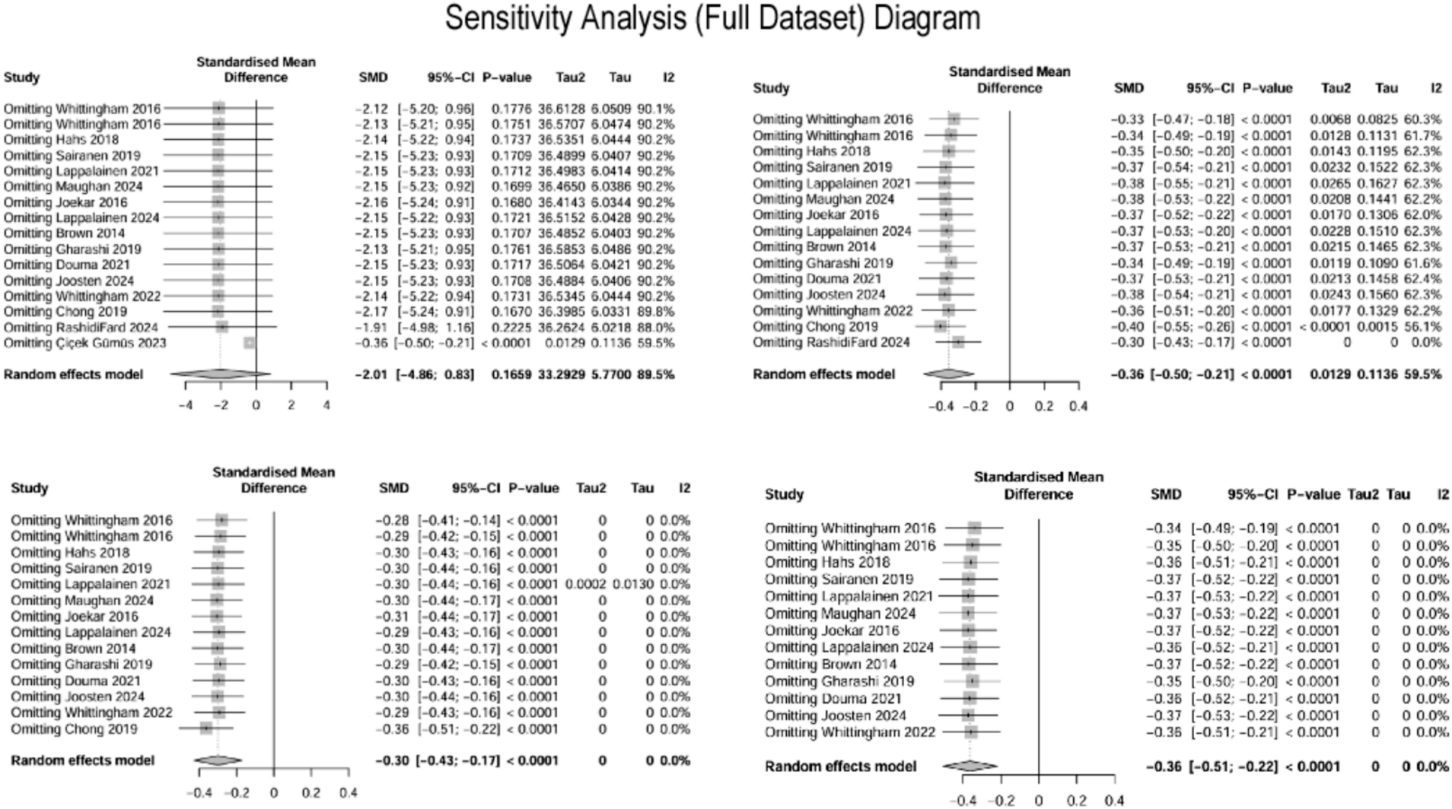
Sensitivity analysis plots.

This study systematically evaluated the intervention effects of ACT on depression in parents of children with special needs by including 15 studies (Brown et al., 2014; Chong et al., 2019; Çiçek Gümüş and Öncel, 2023; Douma et al., 2021; Gharashi et al., 2019; Hahs et al., 2018; Joekar et al., 2016; Joosten et al., 2024; Lappalainen et al., 2021, 2024; Maughan et al., 2024; RashidiFard et al., 2024; Sairanen et al., 2019; Whittingham et al., 2016, 2022), which assessed depressive outcomes in a total of 837 parents. The heterogeneity among the studies was high (I^2^ = 89.5%, *p* < 0.0001), and a random effects model was employed for the analysis. The initial results indicated that ACT did not have a significant effect on depression in parents of children and adolescents with special needs (SMD = −2.01, 95% CI −4.86 ~ 0.83, *p* = 0.166). Given the high heterogeneity (I^2^ > 50%, I^2^ = 89.5%, *p* < 0.0001), Egger’s regression was conducted to evaluate potential publication bias. Egger’s regression, utilized to detect publication bias and assess the influence of minor study effects, initially indicated significant bias (t = −5.06, *p* = 0.000) (Figure 4A), suggesting that minor sample effects or data anomalies may have influenced the results (Gillespie et al., 2012). A sensitivity analysis was conducted to identify the sources of publication bias. This analysis aimed to assess the robustness and reliability of the results by altering key parameters or excluding specific studies. Initially, it was found that the study by Çiçek Gümüş and Öncel (2023) significantly influenced publication bias (Figure 5, top left). Even after excluding this study, Egger’s test indicated significant bias (t = −3.74, *p* = 0.002) (Figure 4B). Further sensitivity analysis revealed that the study by RashidiFard et al. (2024) also substantially contributed to the bias (Figure 5, top right); after excluding this study, Egger’s test remained significant (t = −2.8, *p* = 0.016) (Figure 4C). Moreover, the leave-one-out sensitivity analysis using R software dramatically reduced heterogeneity (I^2^ = 0), preventing further analysis and suggesting outliers. Subsequently, sensitivity analysis using Stata software indicated that the study by Chong et al. (2019) also significantly contributed to publication bias. After excluding the study by Chong et al. (Figure 5, bottom left and bottom right), Egger’s test became non-significant (t = −1.282, *p* = 0.226) (Figure 4D).

A review of the original texts from these three studies revealed the following:

In the study conducted by Çiçek Gümüş and Öncel (2023), depressive symptoms were evaluated using the DASS-21 scale. The experimental group reduced scores from 18.13 ± 3.58 to 5.47 ± 1.77, while the control group’s score was recorded at 17.57 ± 2.90 post-intervention. Considering that effect sizes for psychological interventions typically range between 0.2 and 0.8, the observed value significantly exceeds this range, indicating the potential presence of anomalous data.

In the research by RashidiFard et al. (2024), parental depression scores decreased from 58.0 at pre-test to 36.61 post-intervention, with a further decline to 24.38 at follow-up (representing a reduction of 58%). In contrast, the control group demonstrated minimal change. Furthermore, the study utilized the BDI-II for assessing depression, while the majority of the included studies employed the DASS scale. This inconsistency in measurement instruments could have introduced bias when applying the SMD for correction.

In the investigation by Chong et al. (2019), despite the relatively large sample size (84 participants in both the experimental and control groups), the standard deviations were recorded as 4.22 and 5.5, respectively. The notable disparity in standard deviations and the large sample size may reflect substantial data variability or the presence of outliers, thereby contributing to bias detection. The results of the standardized residuals and Cook’s distance analyses are provided in the Supplementary material.

**Figure 6:**
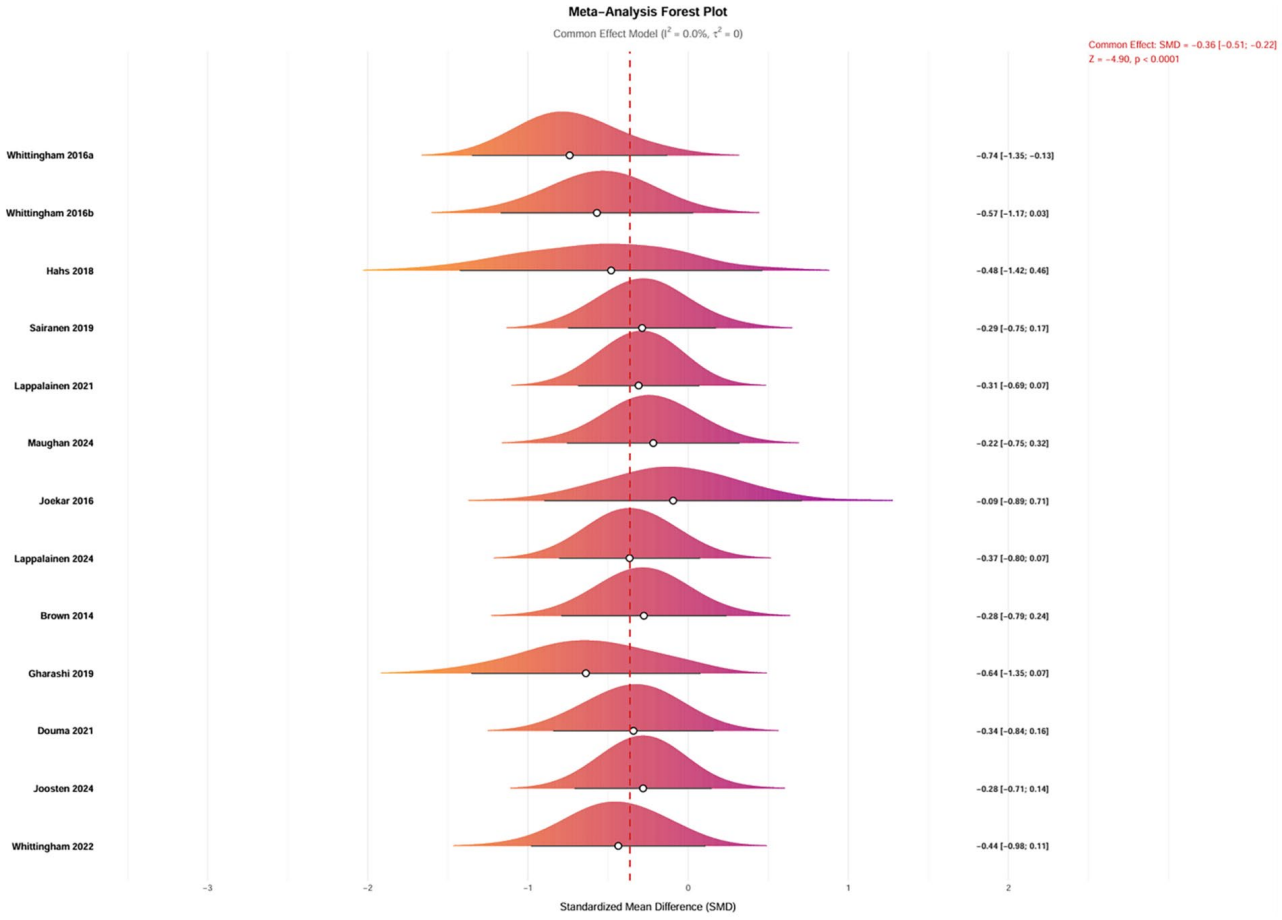
Forest plot of the intervention effects of ACT on depression in parents of children with special needs.

After excluding three studies identified as significant outliers, 12 studies evaluating depressive outcomes in 746 parents were analyzed. The heterogeneity among these studies was low (I^2^ = 0.00%, *p* = 0.99), and a fixed-effects model was employed for the analysis (Figure 6). The findings indicated that ACT had a significant effect on reducing depression in parents of children and adolescents with special needs (SMD = −0.36, 95% CI [−0.51, −0.22], *p* < 0.0001). A random-effects model was also applied to assess the robustness of the results, which remained consistent (SMD = −0.36, 95% CI [−0.51, −0.22], *p* < 0.0001).

### Subgroup analysis

Subgroup analyses were conducted to investigate further the factors influencing the effects of ACT on depression in parents of children with special needs. The analyses considered various factors, including the type of condition affecting children and adolescents, the classification of parents, the modality of intervention, geographic region, duration of intervention, frequency, and cycle. However, no subgroup analysis was performed for parental age, as most parents were between 30 and 40.

**Figure 7:**
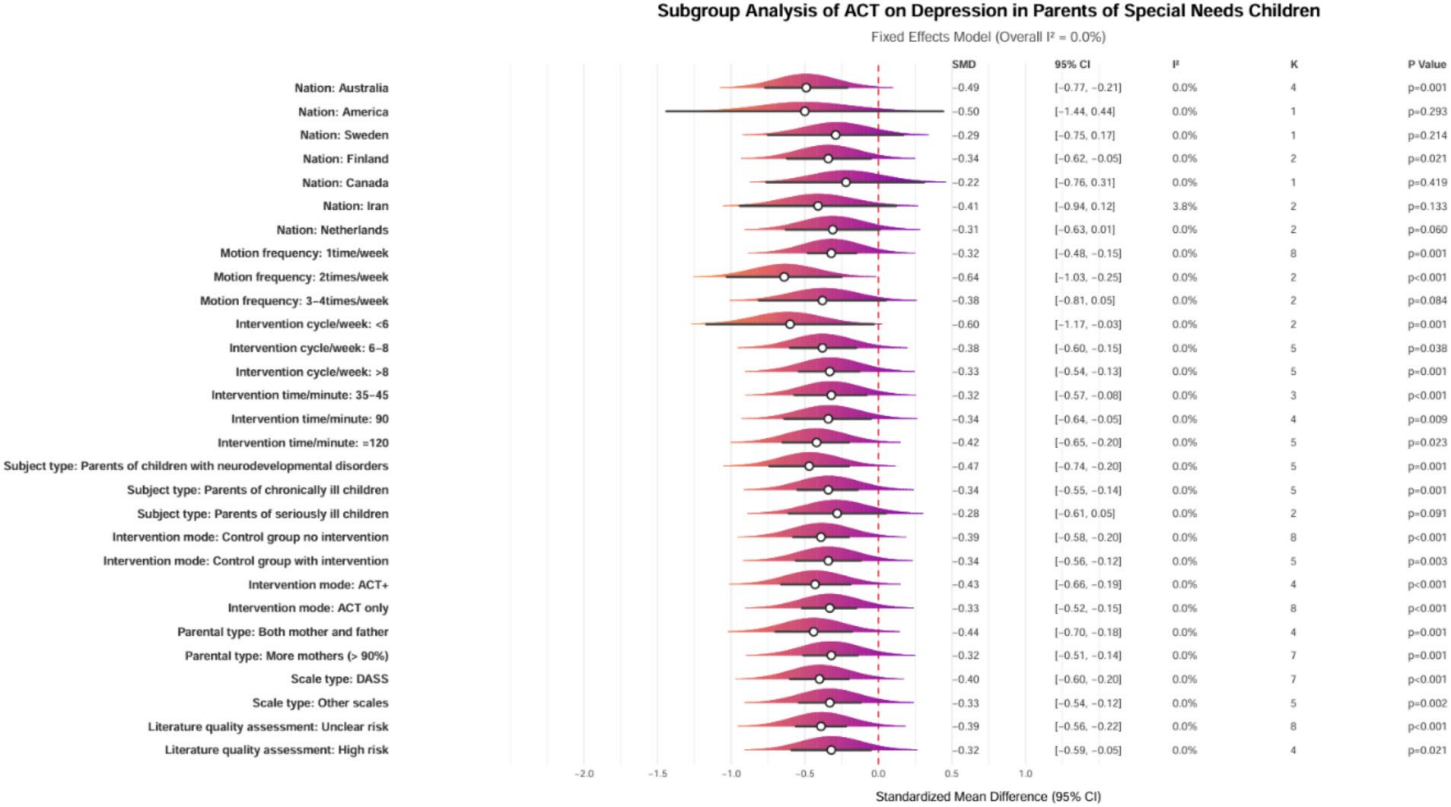
Forest plot of subgroup analysis for the effects of ACT on depression in parents of children with special needs. ACT, acceptance and commitment therapy; SMD, standardized mean difference; CI, Confidence interval; I^2^, I-squared heterogeneity index; K, Number of studies; DASS, depression anxiety stress scales; ACT+, ACT with complementary interventions.

The subgroup analyses revealed heterogeneous characteristics in the effects of ACT on depression among parents of children with special needs. The findings indicated that geographic distribution, intervention parameters, and participant characteristics significantly influenced the effectiveness of the interventions. Specifically, interventions conducted in Australia (SMD = −0.49, 95% CI [−0.77, −0.21], *p* = 0.001) and Finland (SMD = −0.34, 95% CI [−0.62, −0.05], *p* = 0.021) demonstrated significantly better outcomes compared to those in other countries (*p* < 0.05), suggesting that well-developed social welfare systems and cultural adaptability may enhance intervention efficacy. Regarding the design of the intervention, a frequency of 1–2 sessions per week, combined with a session duration of ≥35 min, yielded the optimal effect size (SMD = −0.32 to −0.64, *p* < 0.01). Both short-term interventions (<6 weeks, SMD = −0.60, *p* = 0.001) and long-term interventions (>8 weeks, SMD = −0.33, *p* = 0.001) produced significant effects, although the effect size diminished with prolonged intervention durations. Notably, improvements in depression were significantly more significant among parents of children with neurodevelopmental disorders (SMD = −0.47, *p* = 0.001) and chronic illnesses (SMD = −0.34, *p* = 0.001) than among parents of critically ill children (*p* = 0.091), indicating that interventions should be precisely tailored to the specific type of childhood disease. For parents of children with neurodevelopmental disorders or chronic illnesses, a medium-term ACT intervention consisting of 1–2 sessions per week and a session duration of ≥35 min is recommended in countries with well-developed social welfare systems, such as Australia (Figure 7). Future research should further explore mechanisms for cross-cultural adaptation.

### Meta-regression

**Figure 8:**
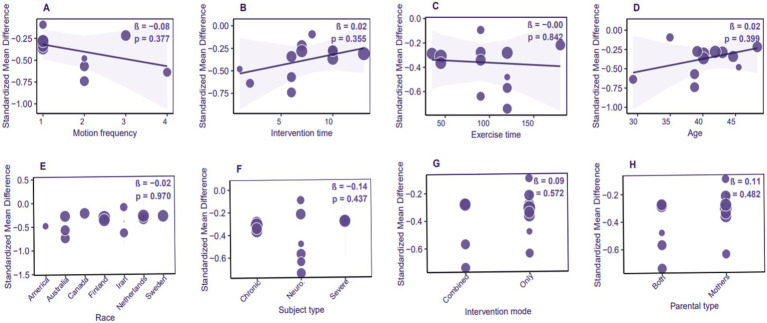
Bubble plot of meta-regression analysis for the effects of ACT on depression in parents of children with special needs.

A meta-regression analysis was conducted utilizing a bubble plot (*n* = 12 studies, I^2^ = 43.2%) to assess the influence of potential moderators on the effects of ACT in alleviating depression among parents of children and adolescents with special needs. The analysis revealed no significant moderating variables; all potential moderators examined did not achieve statistical significance (*p* > 0.05). This includes intervention parameters such as session frequency (*β* = −0.08, *p* = 0.377), intervention duration (*β* = 0.02, *p* = 0.355), and single session duration (*β* = 0.00, *p* = 0.842); demographic characteristics such as parental age (*β* = 0.02, *p* = 0.399) and ethnicity (*β* = −0.02, *p* = 0.970); as well as implementation characteristics including intervention mode (*β* = 0.09, *p* = 0.572) and parental participation type (*β* = 0.11, *p* = 0.482) (Figure 8). The non-significant findings in the meta-regression may be attributed to limited statistical power. It is recommended that future research increase the representation of samples from low- and middle-income countries (as the current studies are concentrated in high-welfare nations) and conduct cross-cultural comparisons.

The subgroup analyses revealed a three-dimensional differential pattern in the effects of ACT on depression among parents of children with special needs:

1. *Geographic heterogeneity:* significant effects were observed in Australia (Standardized Mean Difference [SMD] = −0.49, 95% Confidence Interval [CI] [−0.77, −0.21]) and Finland (SMD = −0.34, 95% CI [−0.62, −0.05]) (*p* < 0.05). These findings suggest that robust social welfare systems may enhance intervention efficacy by alleviating financial burdens on families.
2. *Dose-sensitive window*: high-frequency interventions (1–2 sessions per week) combined with moderate-to-high intensity (single session duration of ≥35 min) yielded optimal effects (SMD = −0.32 to −0.64). Furthermore, short-term intensive interventions (<6 weeks, SMD = −0.60) improved outcomes by 81.8% compared to long-term protocols (SMD = −0.33).
3. *Disease-specific response*: improvements in depression were significantly more excellent among parents of children with neurodevelopmental disorders (SMD = −0.47) and chronic illnesses (SMD = −0.34) compared to those with critically ill children (SMD = 0.19, *p* = 0.091). This reflects differential plasticity in response to psychological interventions based on the disease course.

Although meta-regression did not identify any statistically significant moderators (*p* > 0.05), this may be attributed to the limited sample size in the subgroups (k = 12) and the dilution of single-factor effects by multidimensional interactions. Clinical decisions should primarily rely on subgroup analysis results, emphasizing the importance of the social welfare support system, the dose-sensitive window of the intervention, and the necessity for disease-specific tailoring.

## Discussion

This study confirmed that ACT significantly alleviated depressive symptoms among parents of children with special needs (SMD = −0.36, 95% CI −0.51 to −0.22, *p* < 0.0001). However, its effects were moderated by geographic and cultural contexts, intervention parameters, and the types of children’s illnesses. These results align with the findings of Li et al. (2023), which indicated that ACT-based interventions significantly reduced depressive symptoms in parents of children with special health care needs (SHCN), demonstrating a moderate effect size (Hedges’ g = −0.32). Additionally, further comparative studies revealed that ACT outperformed Behavioral Activation (BA) in mitigating depression and rumination among mothers of children with cerebral palsy, thereby highlighting its exceptional efficacy within this population (Alirahmi et al., 2024). One of the core mechanisms of ACT is the enhancement of psychological flexibility, which is closely associated with reductions in symptoms of depression and anxiety (Zhao et al., 2023). By strengthening psychological skills such as self-compassion and self-adaptation, ACT has improved mental health outcomes among parents (Pyszkowska and Górnik-Durose, 2023). Its mindfulness component allows parents to perceive their situations non-judgmentally, effectively alleviating stress and depressive symptoms (Pots et al., 2016). ACT specifically targets experiential avoidance by reducing parents’ tendencies to escape from negative emotions and thoughts, contributing to the alleviation of depressive symptoms (A-Tjak et al., 2021). Furthermore, the de-centering mechanism of ACT enables parents to view their feelings and thoughts as transient and distinct from the self, thereby mitigating emotional symptoms (Van Eeden, 2024). Group-based ACT sessions incorporating parenting skills have effectively enhanced parental adaptability, improved emotional health, and reduced depressive symptoms (Li et al., 2023). Both online and offline formats of ACT exhibit similar effects, providing greater flexibility in the mode of delivery (Pyszkowska and Górnik-Durose, 2023). Although ACT has achieved promising results in reducing depressive symptoms among parents of children with special health care needs (SHCN), its broader applicability requires further investigation (Frederick et al., 2023). The effectiveness of ACT is not limited to the SHCN population; its successful application in other groups, including postpartum women and young people, further underscores its versatility as a treatment method (Branquinho et al., 2022). However, future research needs to explore the long-term effects of ACT and assess its adaptability across diverse cultural and demographic groups.

Subgroup analyses revealed that ACT significantly alleviated depressive symptoms in parents of children with neurodevelopmental disorders or chronic illnesses, highlighting notable regional differences. In areas with well-established social welfare systems, a medium-term ACT intervention—administered 1–2 times per week, with each session lasting at least 35 min throughout 4–8 weeks—resulted in substantial improvements in depressive symptoms among these parents. Moreover, the effect size exhibited a dose-dependent decrease as the intervention duration was prolonged. This observation aligns with existing literature, which indicates that ACT has a more pronounced effect in mitigating depressive symptoms among mothers of children with cerebral palsy (Alirahmi et al., 2024). Additionally, systematic reviews and meta-analyses further corroborate the conclusion that ACT significantly reduces depression and stress symptoms in parents of children with autism (Li et al., 2024). In resource-limited settings, simplified low-frequency interventions delivered by non-specialists, using evidence-based, low-intensity, transdiagnostic protocols and the strategic deployment of technology, may enhance the scalability and accessibility of ACT (Bockting et al., 2016). ACT has emphasized mindfulness and psychological flexibility, mainly contributing to reducing parental stress and depression. In group settings, ACT interventions have provided a supportive environment where participants can share experiences and learn from one another. Research has demonstrated that group seminars significantly reduce depressive symptoms and improve the mental health of parents with children who have developmental disorders. Furthermore, ACT’s values and committed action components have assisted parents in coping with the emotional challenges associated with their children’s illnesses (Eifert, 2022). Although ACT has shown positive effects in various intervention contexts, some studies have indicated limitations in addressing depression among parents of children with cancer. For instance, an ACT group intervention targeting cancer survivors failed to significantly improve their neuropsychological late effects, suggesting that ACT may encounter similar challenges in alleviating parental depression (Stuart et al., 2023). To comprehensively assess the efficacy of ACT, future research is recommended to employ larger sample sizes and more rigorous designs (Eche et al., 2021). Additionally, regional differences have played a significant role in intervention outcomes. In Australia, for example, enhancing the self-efficacy of key personnel involved in the intervention has effectively supported parental wellbeing, highlighting the importance of parental support networks (Young et al., 2023). Culturally adapted interventions, such as the Family Talk Intervention (FTI) in Finland, have effectively reduced children’s emotional symptoms and improved prosocial behaviors (Solantaus et al., 2010). Although ACT interventions have shown positive effects in several studies, evaluations of their efficacy must consider the broader context of mental health support systems across different countries and regions. To enhance the effectiveness of ACT interventions, it is recommended that they be combined with other supportive services, ensuring that all parents, regardless of socioeconomic status, have equal access to these interventions. Some studies suggest that adequate mental health interventions typically require 4–8 treatment sessions to significantly improve participants’ psychological outcomes (Li et al., 2023). Moreover, the group format is regarded as the preferred mode of intervention, as it facilitates the sharing of experiences and mutual support among participants. This conclusion aligns with the subgroup analysis results of the present study, which supports the effectiveness of a medium-term ACT intervention—administered 1–2 times per week, with each session lasting at least 35 min for 4–8 weeks—in practical applications. The variability in the meta-regression results may be attributed to limited statistical power. Therefore, future research should include more samples from low- and middle-income countries, given the current concentration of studies in high-welfare regions, and conduct cross-cultural comparisons to strengthen generalizability.

## Conclusion

This study confirmed that ACT significantly alleviated depressive symptoms among parents of children with special needs (SMD = −0.36, *p* < 0.0001). However, its effects were influenced by geographic and cultural contexts, intervention parameters, and the types of illnesses affecting the children. The high effect sizes observed in Australia and Finland (SMD = −0.49 to −0.34) suggest that robust social welfare systems may have enhanced the efficacy of the intervention through the mediating role of family support resources. Dose–response analysis indicated a non-linear relationship: a medium-term intervention (4–8 weeks) consisting of sessions lasting at least 35 min, administered 1–2 times per week, emerged as the optimal strategy, although it necessitated dynamic adjustments based on the children’s illness type (neurodevelopmental disorders > chronic illness > severe conditions). Future research should aim to integrate cultural adaptation theories with precision medicine frameworks to optimize the translational pathway of ACT.

### Limitations

The interpretation of the results must be considered in light of specific methodological characteristics. Firstly, while subgroup analyses based on geographic distribution and types of children’s illnesses provided valuable insights into sources of heterogeneity, the variation in reporting exercise intensity parameters across studies may have impacted the precise elucidation of the dose–response relationship. Future research could enhance data comparability by adopting consensus reporting standards, such as the CONSORT guidelines. Secondly, the evaluation of cultural background was limited; thus, future studies could benefit from integrating Hofstede’s cultural dimensions, such as individualism and collectivism, to deepen the understanding of regional effect mechanisms. Additionally, there were limitations in the subgroup analyses concerning identifying factors influencing intervention effects, as the grouping settings imposed certain constraints. Finally, the studies included in this analysis were restricted to controlled trials published in English, potentially leading to the exclusion of relevant literature in other languages. The variability in meta-regression results may be attributed to limited statistical power. It is recommended that future research increase representation from low- and middle-income countries, given the current focus on high-welfare nations, and conduct cross-cultural comparisons to enhance the generalizability of findings.

## Data Availability

The raw data supporting the conclusions of this article will be made available by the authors, without undue reservation.

## Footnotes

^1^ https://www.pymeta.com/evdmap/
