# Supplementary material for: The effect of acceptance and commitment therapy on depression in parents of children with special needs: a meta-analysis
Source: Front Psychol. 2025 Jul 9;16:1590489. doi: 10.3389/fpsyg.2025.1590489 (PMC12283575; doi:10.3389/fpsyg.2025.1590489)
Supplement: Supplementary file 1 [file Supplementary_file_1.docx]

**​​****Table 1.Cochrane Risk of Bias Assessment Table**

| Author/Year | Random Sequence Generation | Allocation Concealment | Blinding of Participants | Blinding of Personnel | Blinding of Outcome Assessment | Incomplete Outcome Data | Selective Reporting | Other Bias | Overall Risk |
| --- | --- | --- | --- | --- | --- | --- | --- | --- | --- |
| Whittingham et al. (2016a) | Low risk | Unclear risk​​ | Unclear risk​​ | Low risk | Low risk | Unclear risk​​ | Unclear risk​​ | Low risk | Unclear risk​​ |
| Whittingham et al. (2016b) | Low risk | Unclear risk​​ | Unclear risk​​ | Low risk | Low risk | Unclear risk​​ | Low risk | Unclear risk​​ | Unclear risk​​ |
| Hahs et al. (2018) | Low risk | Low risk | Unclear risk​​ | Low risk | Low risk | Low risk | Unclear risk​​ | Unclear risk​​ | Unclear risk​​ |
| Sairanen et al. (2019) | Low risk | Unclear risk​​ | High risk | Unclear risk​​ | Unclear risk​​ | Unclear risk​​ | Low risk | Unclear risk​​ | High risk |
| Lappalainen et al. (2021) | Low risk | Low risk | Unclear risk​​ | Low risk | Unclear risk​​ | Low risk | Low risk | Low risk | Unclear risk​​ |
| Maughan et al. (2024) | Low risk | Low risk | Unclear risk​​ | Low risk | Low risk | Low risk | Low risk | Unclear risk​​ | Unclear risk​​ |
| Joekar et al. (2016) | Low risk | Unclear risk​​ | Unclear risk​​ | Low risk | Low risk | Low risk | High risk | Low risk | High risk |
| Lappalainen et al. (2024) | Low risk | Unclear risk​​ | Unclear risk​​ | Unclear risk​​ | Unclear risk​​ | Unclear risk​​ | Unclear risk​​ | Unclear risk​​ | Unclear risk​​ |
| Brown et al. (2014) | Low risk | Low risk | Unclear risk​​ | Unclear risk​​ | Low risk | Low risk | Low risk | Low risk | Unclear risk​​ |
| Gharashi et al. (2019) | Low risk | High risk | Unclear risk​​ | Unclear risk​​ | Unclear risk​​ | Low risk | High risk | Unclear risk​​ | High risk |
| Douma et al. (2021) | Low risk | Low risk | Unclear risk​​ | Low risk | Unclear risk​​ | Unclear risk​​ | Unclear risk​​ | Low risk | Unclear risk​​ |
| Joosten et al. (2024) | Low risk | Unclear risk​​ | Unclear risk​​ | High risk | Unclear risk​​ | Low risk | Low risk | Low risk | High risk |
| Whittingham et al. (2022) | Low risk | Low risk | Unclear risk​​ | Unclear risk​​ | Low risk | Unclear risk​​ | Unclear risk​​ | Low risk | Unclear risk​​ |
| Chong et al. (2019) | Low risk | Unclear risk​​ | Unclear risk​​ | Unclear risk​​ | Low risk | Low risk | Unclear risk​​ | Unclear risk​​ | Unclear risk​​ |
| RashidiFard et al. (2024) | Low risk | Unclear risk​​ | Unclear risk​​ | High risk | Unclear risk​​ | Low risk | Unclear risk​​ | Low risk | Unclear risk​​ |
| Çiçek Gümüş. (2023) | Low risk | Unclear risk​​ | High risk | Unclear risk​​ | Unclear risk​​ | Unclear risk​​ | Unclear risk​​ | Unclear risk​​ | High risk |


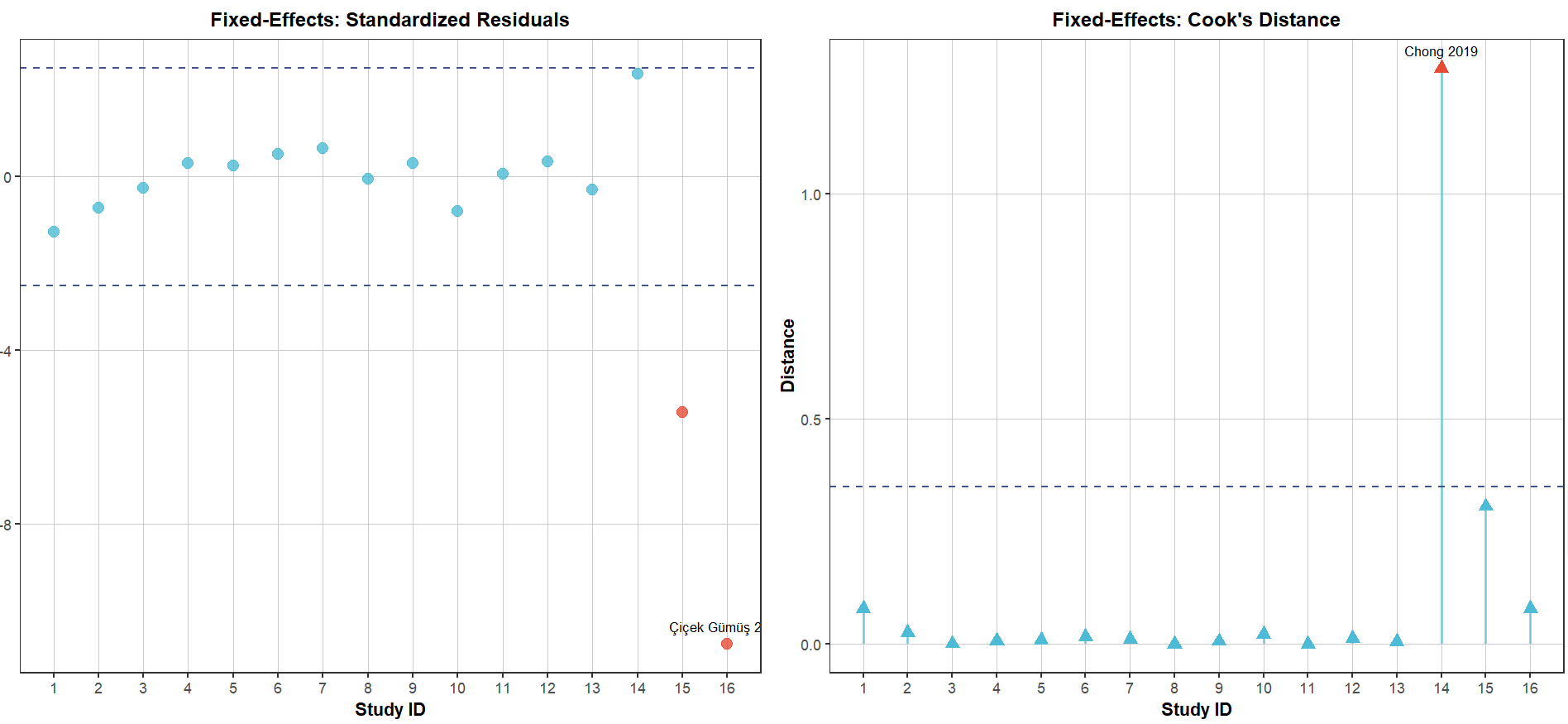


**​Outlier Exclusion Analysis​**​
Three studies were identified as outliers based on:

- Standardized residuals > ±2.5
- Cook's distance > 3×mean value

**Table 2.​**​ Impact of Sequential Outlier Exclusion on Pooled Effect Size

| **Model** | **SMD (95% CI)** | **I²** |
| --- | --- | --- |
| Including all outliers | -2.01 (-4.86, 0.83) | 89.50% |
| Excluding Chong et al. | -2.16 (-5.24, 0.91) | 89.80% |
| Excluding RashidiFard & Chong | -2.07 (-5.40, 1.26) | 88.40% |
| Excluding all three outliers | -0.36 (-0.51, -0.22) | 0.00% |

**Table 3. Subgroup Analysis Results of Acceptance and Commitment Therapy (ACT) on Depression in Parents of Special Needs Children**

| Dimensionality | sort | Number of studies/papers | I^2^ | Effect model | SMD and 95%CI | P |
| --- | --- | --- | --- | --- | --- | --- |
| Nation | Australia | 4 | 0.0% | Fix | -0.49(-0.77,-0.21) | 0.001 |
|  | America | 1 | 0.0% | Fix | -0.50(-1.44,0.44) | 0.293 |
|  | Sweden | 1 | 0.0% | Fix | -0.29(-0.75,0.17) | 0.214 |
|  | Finland | 2 | 0.0% | Fix | -0.34(-0.62,-0.05) | 0.021 |
|  | Canada | 1 | 0.0% | Fix | -0.22(-0.76,0.31) | 0.419 |
|  | Iran | 2 | 3.8% | Fix | -0.41(-0.94,0.12) | 0.133 |
|  | Netherlands | 2 | 0.0% | Fix | -0.31(-0.63,0.01) | 0.060 |
| Motion frequency | 1time/week | 8 | 0.0% | Fix | -0.32(-0.48,-0.15) | 0.001 |
|  | 2times/week | 2 | 0.0% | Fix | -0.64(-1.03,-0.25) | 0.000 |
|  | 3-4times/week | 2 | 0.0% | Fix | -0.38(-0.81,0.05) | 0.084 |
| Intervention cycle/week | ＜6 | 2 | 0.0% | Fix | -0.60(-1.17,-0.03) | 0.001 |
|  | 6-8 | 5 | 0.0% | Fix | -0.38(-0.60,-0.15) | 0.038 |
|  | ＞8 | 5 | 0.0% | Fix | -0.33(-0.54,-0.13) | 0.001 |
| Intervention time/minute | 35-45 | 3 | 0.0% | Fix | -0.32(-0.57,-0.08) | 0.000 |
|  | 90 | 4 | 0.0% | Fix | -0.34(-0.64,-0.05) | 0.009 |
|  | ≥120 | 5 | 0.0% | Fix | -0.42(-0.65,-0.20) | 0.023 |
| Subject type | Parents of children with neurodevelopmental disorders | 5 | 0.0% | Fix | -0.47(-0.74,-0.20) | 0.001 |
|  | Parents of chronically ill children | 5 | 0.0% | Fix | -0.34(-0.55,-0.14) | 0.001 |
|  | Parents of seriously ill children | 2 | 0.0% | Fix | -0.28(-0.61,0.05) | 0.091 |
| Intervention mode | Control group no intervention | 8 | 0.0% | Fix | -0.39(-0.58,-0.20) | 0.000 |
|  | Control group with intervention | 5 | 0.0% | Fix | -0.34(-0.56,-0.12) | 0.003 |
|  | ACT+ | 4 | 0.0% | Fix | -0.43(-0.66,-0.19) | 0.000 |
|  | ACT only | 8 | 0.0% | Fix | -0.33(-0.52,-0.15) | 0.000 |
| Parental type | Both mother and father | 4 | 0.0% | Fix | -0.44(-0.70,-0.18) | 0.001 |
|  | More mothers (> 90%) | 7 | 0.0% | Fix | -0.32(-0.51,-0.14) | 0.001 |
| Scale type | DASS | 7 | 0.0% | Fix | -0.40(-0.60,-0.20) | 0.000 |
|  | Other scales | 5 | 0.0% | Fix | -0.33(-0.54,-0.12) | 0.002 |
| Literature quality assessment | Unclear risk | 8 | 0.0% | Fix | -0.39(-0.56,-0.22) | 0.000 |
|  | High risk | 4 | 0.0% | Fix | -0.32(-0.59,-0.05) | 0.021 |

**Retrieval Strategy**

**1. PubMed​​**

("Acceptance and Commitment Therapy"[Mesh] OR "ACT"[tiab] OR "acceptance and commitment therapy"[tiab])

AND

("Depression"[Mesh] OR "Depressive Disorder"[Mesh] OR depress*[tiab] OR "depressive symptoms"[tiab])

AND

("Parents"[Mesh] OR "Caregivers"[Mesh] OR parent*[tiab] OR mother*[tiab] OR father*[tiab] OR caregiver*[tiab])

AND

("Disabled Children"[Mesh] OR "Autism Spectrum Disorder"[Mesh] OR "Cerebral Palsy"[Mesh] OR "Chronic Disease"[Mesh] OR "Brain Injuries, Traumatic"[Mesh] OR "Hearing Loss"[Mesh] OR "Asthma"[Mesh] OR "Neoplasms"[Mesh] OR "special needs children"[tiab] OR "children with chronic illness"[tiab] OR "children with autism"[tiab])

AND

("Randomized Controlled Trial"[pt] OR "Controlled Clinical Trial"[pt] OR "Clinical Trial"[pt] OR random*[tiab] OR "control group"[tiab] OR "RCT"[tiab])

NOT

("Review"[pt] OR "Case Reports"[pt] OR "Comment"[pt])

**2. Cochrane Library**

#1 [mh "Acceptance and Commitment Therapy"] OR "ACT":ti,ab,kw OR "acceptance and commitment therapy":ti,ab,kw

#2 [mh Depression] OR [mh "Depressive Disorder"] OR depress*:ti,ab,kw OR "depressive symptoms":ti,ab,kw

#3 [mh Parents] OR [mh Caregivers] OR parent*:ti,ab,kw OR mother*:ti,ab,kw OR father*:ti,ab,kw

#4 [mh "Disabled Children"] OR [mh "Autism Spectrum Disorder"] OR [mh "Cerebral Palsy"] OR [mh "Chronic Disease"] OR "special needs children":ti,ab,kw OR "children with autism":ti,ab,kw OR "children with cerebral palsy":ti,ab,kw

#5 [mh "Randomized Controlled Trials"] OR "randomized controlled trial":ti,ab,kw OR RCT:ti,ab,kw OR "controlled trial":ti,ab,kw

#6 #1 AND #2 AND #3 AND #4 AND #5

Publication Date to 15 April 2025, Language: English

**3. Web of Science**

TS=("acceptance and commitment therapy" OR ACT)

AND

TS=(depress* OR "depressive disorder*" OR "mood disorder*")

AND

TS=(parent* OR mother* OR father* OR caregiver*)

AND

TS=("special needs children" OR "children with autism" OR "children with cerebral palsy" OR "children with chronic illness" OR "disabled children" OR "autism spectrum disorder" OR "chronic disease" OR "brain injury" OR "hearing impairment" OR "asthma" OR "cancer")

AND

TS=("randomized controlled trial" OR RCT OR "controlled trial" OR "clinical trial")

NOT

TS=("review" OR "case report" OR "commentary")

Refined by: LANGUAGE: (English) AND DOCUMENT TYPES: (ARTICLE)

**4. PsycINFO**

1. exp "Acceptance and Commitment Therapy"/ OR ACT.mp. OR "acceptance and commitment therapy".ti,ab.

2. exp Depression/ OR depress*.ti,ab. OR "depressive symptom*".ti,ab.

3. exp Parents/ OR exp Caregivers/ OR parent*.ti,ab. OR mother*.ti,ab. OR father*.ti,ab.

4. exp Disabled Children/ OR exp Autism Spectrum Disorders/ OR "cerebral palsy".ti,ab. OR "chronic disease*".ti,ab. OR "special needs children".ti,ab.

5. exp Randomized Controlled Trials/ OR random*.ti,ab. OR "control group".ti,ab. OR RCT.ti,ab.

6. 1 AND 2 AND 3 AND 4 AND 5

7. limit 6 to (yr<=2025 and english language and journal article and human and adulthood (18 plus years))

8. remove duplicates from 7
